# El Niño-Southern Oscillation: a call to action for public health emergency preparedness and response

**DOI:** 10.1016/j.lana.2023.100601

**Published:** 2023-09-21

**Authors:** Esteban Ortiz-Prado, Alex Camacho-Vasconez, Juan S. Izquierdo-Condoy, Celso Bambaren, Leonardo Hernández-Galindo, Juan Carlos Sanchez

**Affiliations:** a One Health Research Group, Faculty of Health Science, Universidad de Las Américas, Calle de los Colimes y Avenida De los Granados, Quito, 170137, Ecuador; b Health Emergencies Department, Pan American Health Organization/World Health Organization, 525 23rd St N.W., Washington, DC, 22037, United States

The public health implications of the ongoing El Niño-Southern Oscillation (ENSO) events in 2023 are significant. The primary health consequences related to El Niño, such as malnutrition, heat stress-respiratory diseases, vector and waterborne diseases, direct injuries and fatalities, disruption of health services, and mental health effects, among others, should be broadly addressed considering local contexts and vulnerabilities, with a multi-hazard management approach.^1^

Recent research and studies have highlighted the patterns of inter-annual climate variability associated with El Niño-Southern Oscillation (ENSO). These patterns can lead to climatic and environmental anomalies that favor the outbreak and spread of various diseases, posing a significant risk to health systems globally.^2^

During the intense 2015–2016 El Niño event, a comprehensive analysis of disease outbreaks was conducted, revealing significant correlations between climatic anomalies and disease incidence in multiple El Niño-connected regions around the world. Changes in precipitation, temperature, and vegetation, characterized by both drought and excessive flooding, created ideal conditions for the emergence and spread of disease vectors.^3^ This led to clusters of disease activity in regions such as Southeast Asia, Tanzania, the western USA, and Brazil, where outbreaks of chikungunya, hantavirus, Rift Valley fever, cholera, plague, and Zika were observed.^4^ Additionally, regions experiencing higher rainfall faced a greater risk of vector-borne diseases, such as dengue, malaria, and Zika, as well as diarrheal diseases and drowning due to overflowing rivers.^5^ Irregular migrants moving through Latin America and the Caribbean are particularly vulnerable due to infections and lack of access to healthcare; in 2023, over 250,000 people traversed the Darien jungle on foot. Of this number, 40,000 were children, and half of them were under the age of five.^6^ These children have unique needs and are at a significant risk of contracting diarrheic and respiratory infections. Additionally, they are exposed to environmental hazards such as flooding, extreme temperatures, and heavy rainfall, intensified by the effects of El Niño. These conditions significantly amplify the dangers during their journey to the northern part of the American continent. The inherent perils of traveling hundreds of miles through the jungle present increased risks of vector-borne and food or water-related diseases.^7^

Furthermore, it has been evidenced that the intensity of disease activity in ENSO tele connected regions was notably higher (approximately 2·5–28%) during years with El Niño events compared to years without. Historically, major El Niño events have triggered widespread and catastrophic environmental and health impacts.^2^ These include increased transmission of infectious diseases due to environmental changes, alterations in population density, and disruptions in public health services. Malaria has experienced a global increase, with significant epidemics reported during past El Niño events.^2^ Similarly, waterborne diseases, such as leptospirosis and cholera, have been intensified by heavy rains and abrupt climatic shifts, leading to considerable fatalities and injuries among children but also adults in countries like Bolivia, Ecuador, Paraguay, and Peru.^8^^,^^9^

In 2023, El Niño notably affected health infrastructure in various countries, particularly in South America. In nations such as Peru and Ecuador, health facilities underwent significant physical damage due to El Niño, requiring the allocation of considerable resources for repairs and reconstruction.^8^ Nonetheless, it's imperative to understand that many of these health infrastructure challenges can be predicted and resolved through adept urban planning, design, and safe facility construction, along with the execution of Resilient Hospital programs.^10^ Elements such as exposure, structural components, and lifelines are crucial for safeguarding health workers, patients, and families, minimizing damages to infrastructure and equipment, and ensuring health facilities persist in delivering high-quality healthcare.^11^

Given the ongoing impacts of the 2023 El Niño phenomenon, a cohesive and rapid response involving stakeholders such as local governments, international organizations, and funding agencies is crucial. Proactive health emergency and disaster risk management with a one-health perspective can enable sectors to coordinate, plan, and consistently refine their preparedness strategies. Local administrations should prioritize building resilient communities, incorporating ancestral knowledge, articulating risks, and collectively bolstering health infrastructure.

Effective early warning and forecasting systems, facilitated by meteorological institutions and risk management entities, are pivotal for the prevention, mitigation, and response in healthcare systems, considering the potential direct and indirect health repercussions of the phenomenon. International organizations and funding entities have a crucial role in collaborating with affected nations to adapt interventions, disseminate knowledge, and assure the unbroken provision of health services.

Facing the profound repercussions of the 2023 El Niño phenomenon demands more than recognition; it requires immediate, coordinated action. Every moment delayed risks countless lives and deteriorating health conditions across the globe. We urge local governments, international organizations, and funding entities to consolidate their efforts, amplify the urgency of the situation, and allocate resources with a renewed sense of purpose. The imperative now is not only to react but to proactively strategize, share intelligence, and fortify our global health infrastructure against these impending challenges. The continuity of health services and the welfare of countless individuals hang in the balance.

## Contributors

Conceptualization: EOP, JSIC; methodology: EOP, ACV, JSIC; resources: All authors; software: All authors; validation: CB, LHG, and JCS; formal analysis: EOP, JSIC; writing—original draft preparation: EOP, JSIC; writing—review and editing: ACG, CB, LHG, JCS; visualization: EOP, ACV, JSIC, CB, LSG. All authors have read and accepted the published version of the manuscript.

## Declaration of interests

The authors declare no conflict of interest.
